# Changes in the Surface Texture of Thermoplastic (Monomer-Free) Dental Materials Due to Some Minor Alterations in the Laboratory Protocol—Preliminary Study

**DOI:** 10.3390/ma15196633

**Published:** 2022-09-24

**Authors:** Bozhana Chuchulska, Ilian Hristov, Boyan Dochev, Raycho Raychev

**Affiliations:** 1 Department of Prosthetic Dental Medicine, Faculty of Dental Medicine, Medical University of Plovdiv, 4000 Plovdiv, Bulgaria; 2 Department of Mechanics, Faculty of Mechanical Engineering, Technical University of Sofia, Branch Plovdiv, 4000 Plovdiv, Bulgaria

**Keywords:** thermoplastic materials, laboratory protocol, dentures, texture, roughness

## Abstract

Contemporary thermoplastic monomer-free prosthetic materials are widely used nowadays, and there are a great variety available on the market. These materials are of interest in terms of the improvement of the quality features of the removable dentures. The aim of this study is to establish how minimal changes in the laboratory protocol of polyamide prosthetic base materials influence the surface texture. Two polyamide materials intended for the fabrication of removable dentures bases were used—Perflex Biosens (BS) and VertexTM ThermoSens (TS). A total number of 20 coin-shaped samples were prepared. They were injected under two different modes—regular, as provided by the manufacturer, and modified, proposed by the authors of this study. Scanning electronic microscopy (SEM) under four magnifications—×1000, ×3000, ×5000, and ×10,000—was conducted. With minimal alterations to the melting temperature (5 °C) and the pressure (0.5 Bar), in Biosens, no changes in terms of surface improvement were found, whereas in ThermoSens, the surface roughness of the material significantly changed in terms of roughness reduction. By modifying the technological mode during injection molding, a smoother surface was achieved in one of the studied materials.

## 1. Introduction

The quality and efficiency of prosthetic treatments depend on the properties of the base prosthetic materials. It is often the case that with removable dentures, complications occur, e.g., denture stomatitis, caused by microflora with various degrees of virulence. Dental prostheses are potential sites of adsorption and colonization of various microorganisms. One of the conditions determining the degree of bacterial adhesion and colonization resistance is the surface structure of the base material.

Denture surface can be affected by various mechanisms. Such mechanisms may include aging and wear and tear [1]. Professional hygienic and cleaning procedures, as well as the instruments used during these procedures, increase the roughness of the material and the risk of future bacterial or fungal contamination [2]. Substantial changes in the surface morphology, increased hydrophilicity and higher optical density of the adhered microorganisms are observed when various chemical agents are used for denture cleaning [3].

Studying at a molecular level the correlation between the surface of the restorative material and the microorganisms in the oral cavity, G. Allias concluded that a conditio sine qua non for micro-floral contamination is related to the material’s texture and depends on the surface tension [4]. The higher the surface tension, the higher the probability for pathogenic microbial contamination is. The surface tension of a given material depends on the material’s technology and processing algorithm, as well as the inclusion of other materials over the prosthetic material’s surface that alter the surface tension [5].

The most common pathogen causing denture stomatitis is *C. albicans* [6]. *C. albicans*, as a conditionally pathogenic species, can asymptomatically colonize both the surfaces of the denture and the mucosa [7]. Al-Dwairi emphasized the significance of *Candida* spp. isolated from the fingertips of removable denture wearers as a source of re-infection of the oral cavity [8]. L. Gendreau identified the spread of denture stomatitis in approximately 70% of the removable denture wearers, and the frequency is higher in elder patients of the female gender [9].

Conventional acrylic resin exhibits highly hydrophilic properties and solubility [10], as well as heterogeneity of the surface texture, further causing internal and surface tension and the formation of cavities where microorganisms infiltrate and propagate. This leads to the disturbance of the micro-biocenosis in the oral cavity, inflammation of the mucosa beneath the denture and the development of denture stomatitis of various etiologies [11].

Nowadays, a great variety of prosthetic materials are available on the market. However, the issue of their interaction with the oral microflora, as well as how the microflora affects these materials, is still understudied. Therefore, the correlation between the microfloral adhesion to the various prosthetic materials and their texture remains a topical question, as does the search for solutions for the improvement of the microstructure and degree of roughness of these materials.

Contemporary thermoplastic monomer-free prosthetic materials are of interest in terms of the improvement of the quality features of removable dentures. However, they are still not sufficiently explored regarding their microbial contamination and colonization. Reliable information can be obtained by performing microbiological and high-magnification microscopic studies in parallel. This would allow for exploring the structures at a nano level. The purpose of this study is to establish how minor alterations in the laboratory protocol of polyamide prosthetic base materials influence the surface texture of these materials.

## 2. Materials and Methods

### 2.1. Materials and Samples

**Figure 1:**
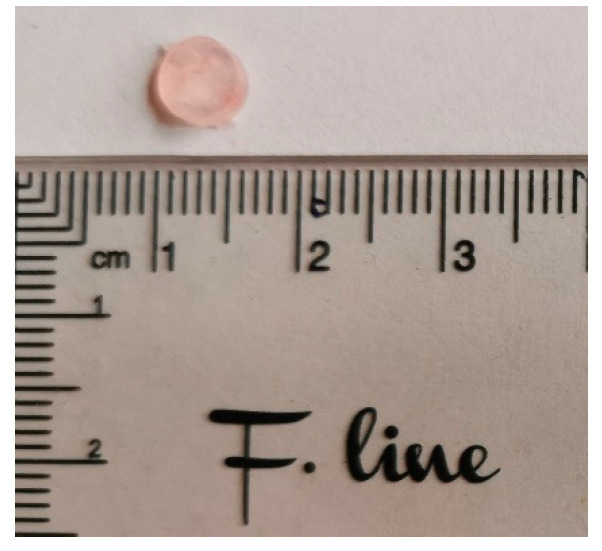
Test samples for observation.

In this study, two polyamide materials intended for the fabrication of removable dentures were used—Perflex Biosens (BS) and VertexTM ThermoSens (TS). A total number of 20 coin-shaped samples were prepared with a diameter of 5 mm and 1 mm thickness (Figure 1).

### 2.2. Methods

#### 2.2.1. Technological Mode

The samples were injected under two different modes—regular, as provided by the manufacturer (Table 1), and modified, proposed by the authors of this study (Table 2). Ten samples of the two tested materials were injected under the regular technological mode, and the other ten samples, five of each material, were injected under a modified mode.

#### 2.2.2. Scanning Electronic Microscopy (SEM)

**Figure 2:**
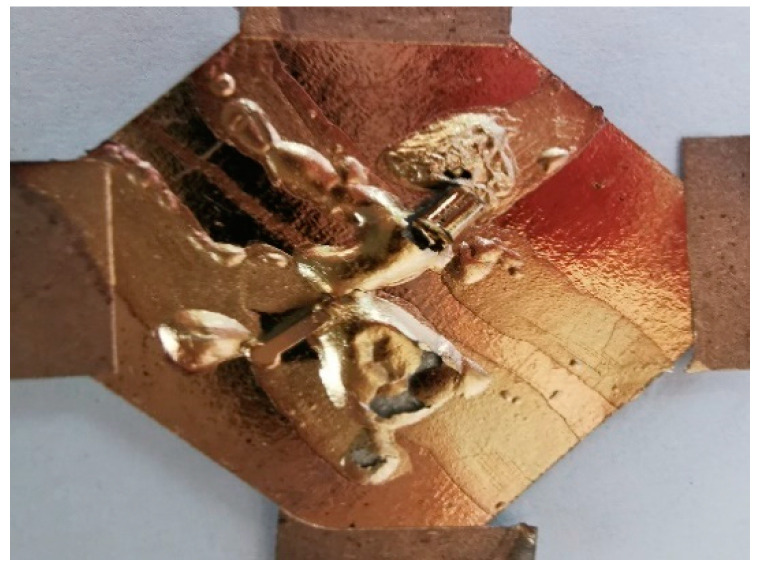
Samples ready for scanning.

The test samples from the two polyamide materials, under the two different technological modes, were plated in 24-carat gold powder (Figure 2) and were scanned using SEM in four different magnifications: ×1000, ×3000, ×5000, and ×10,000.

#### 2.2.3. Microbiological Evaluation

Microbiological evaluation of mucosal and denture surface samples was performed. Samples were collected by swabbing and transported to the laboratory of microbiology within the same day. Swabs were cultured on Sabouraud-dextrose agar (SDA) and incubated for up to 48 h at 30 °C. Colony identification was performed by using matrix-assisted laser desorption time-of-flight mass spectrometry (MALDI-TOF MS, Vitek MS, bioMerieux, Craponne, France). Samples were stained with Löffler methylene blue and observed using ×100 immersion oil microscopy.

## 3. Results

### 3.1. Samples under Regular Technological Mode

**Figure 3:**
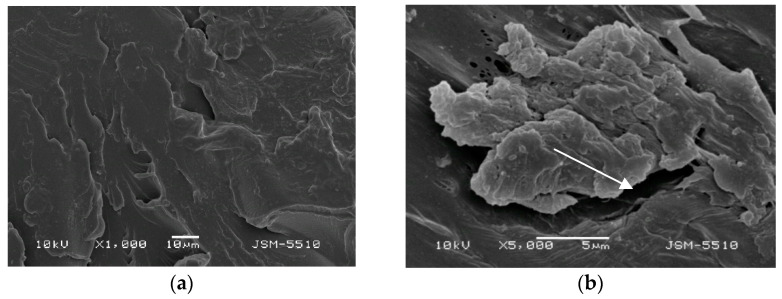
(**a**) BS under magnification ×1000. (**b**) BS under magnification ×5000.

**Figure 4:**
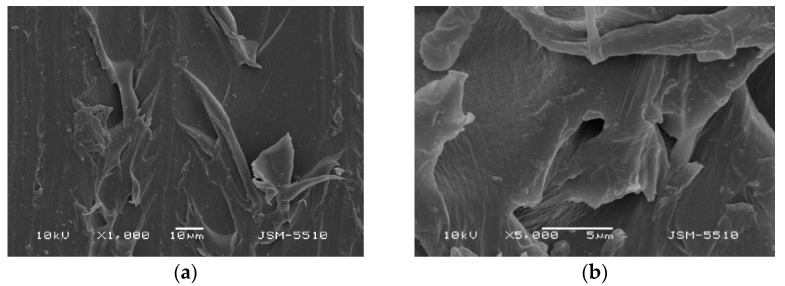
(**a**) TS under magnification ×1000. (**b**) TS under magnification ×5000.

The investigation with SEM methods of the samples injected under the regular technological mode showed different types of defects and numerous spots of unevenness on the surface of both materials under all magnifications. (Figure 3a,b and Figure 4a,b).

#### 3.1.1. Samples Made of Biosens

On the surface of the BS test samples, holes, openings, deep grooves, caverns, and some areas of a rough surface resembling orange peel can be observed. In the ×5000 magnification photo, the dimensions of these surface defects can be measured, and they vary a lot. Portions of the surface display a mica-like texture.

#### 3.1.2. Samples Made of Thermosens

On the TS surface, expressed unevenness with openings, grooves, caverns and a mica-like surface can be observed, along with some bulging formations and deep and undermined areas, and at some points surface destruction can be observed. In the ×5000 magnification photo, it can be observed that these defects form undermined and predilection zones for the retention of different microorganisms.

### 3.2. Samples under Modified Technological Mode

#### 3.2.1. Samples Made of Biosens

**Figure 5:**
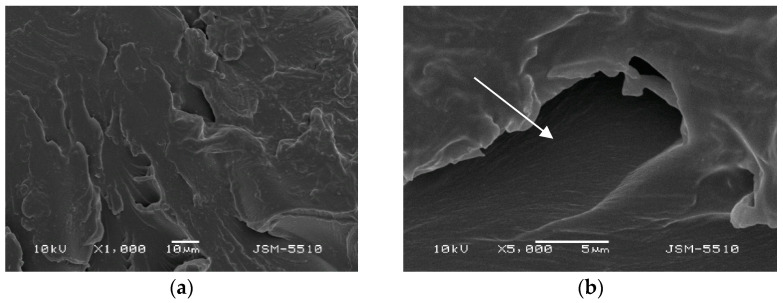
(**a**) BS under magnification ×1000. (**b**) BS under magnification ×5000.

Observations of the surface of BS samples prepared under the modified technological mode do not show any significant differences in the defects compared to the test samples injected under optimal fabrication parameters. Under ×1000 magnification, slight smoothing of the texture is observed; however, the mica-like surface remains unchanged, and the presence of openings and canals is clearly visible (Figure 5a,b). A magnification of ×5000 reveals that these openings grow into deep caverns more than 20 microns in size.

#### 3.2.2. Samples Made of Thermosens

**Figure 6:**
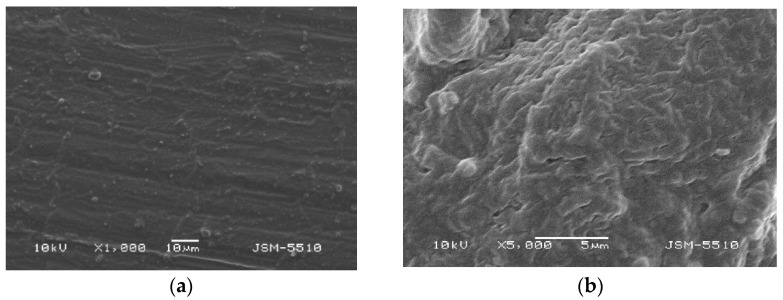
(**a**) TS under magnification ×1000. (**b**) TS under magnification ×5000.

Observations of the surface of TS test samples prepared under the modified technological mode show significant differences in the surface characteristics compared to the test samples injected under the optimal technological mode. Under ×1000 magnification, smoothening of the texture is observed, where shallow grooves and unevenness with a bubble-like shape can be seen; the structure is slightly wavy (Figure 6a,b). Under a magnification of ×5000, the surface is orange peel-textured; however, the uneven areas and deep defects do not exceed 1–3 microns. It should be noted that the refinement of the surface texture of this material is a direct result of the technological mode modification, but the effect on the mechanical properties has yet not been investigated.

Although the form of the defects is too complex to be measured precisely, some dimensions are given in the following table (Table 3).

**Figure 7:**
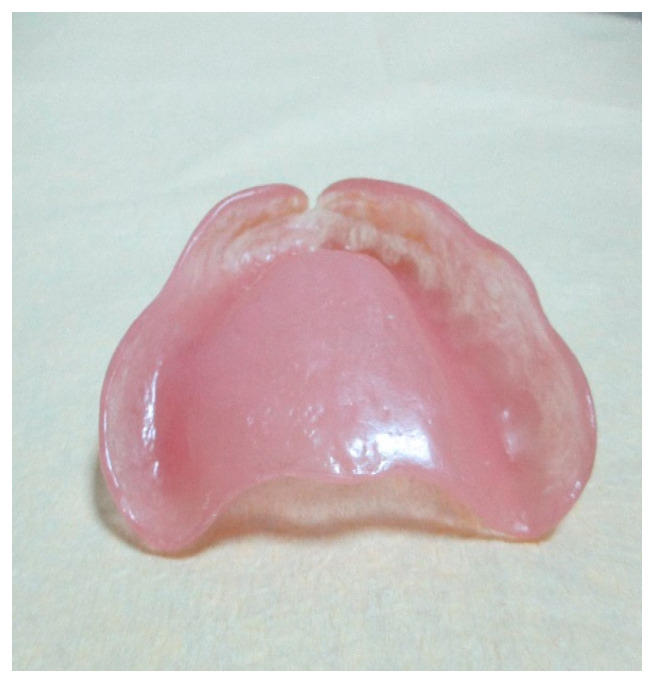
Patient’s denture made from ThermoSens using the conventional laboratory method.

Ten patients were included in this pilot study. Five Thermosens dentures and five Biosens dentures were created. The patients were examined during regular (every two weeks) follow-ups. Two of them (one male, 72 years old and one female patient, 69 years old) showed clinical symptoms of denture stomatitis (Figure 7).

**Figure 8:**
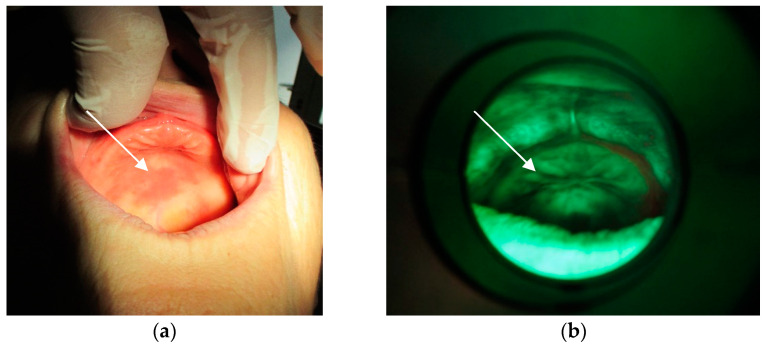
(**a**) Mucosa with lesions. (**b**) Fungal colonies over the mucosa.

**Figure 9:**
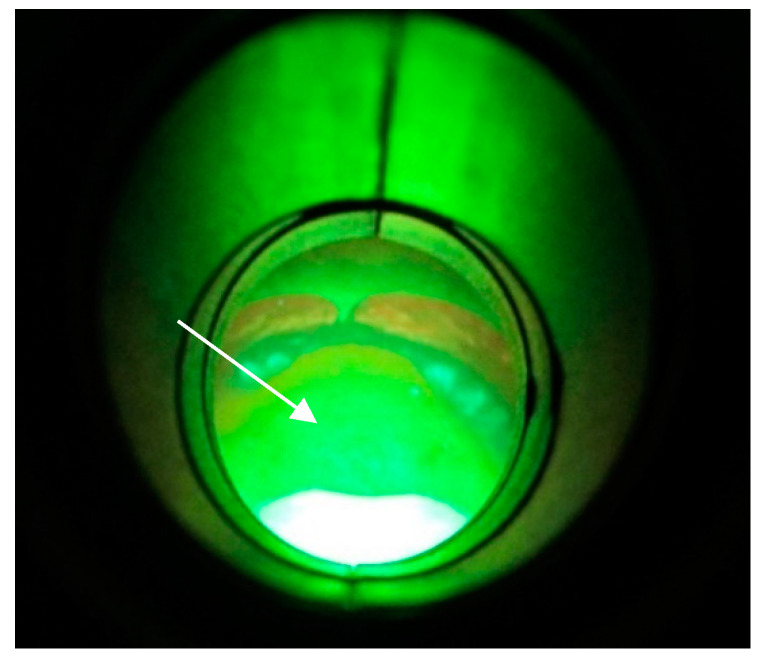
Colonized ThermoSens denture.

The method of direct fluorescence visualization with the help of the VELscope^®^ (LED Dental, Inc., White Rock, BC, Canada) device was applied. Contamination not only of the mucosa (Figure 8a,b) beneath the denture but also on the denture surface itself was ascertained (Figure 9).

The symptoms started at the end of the sixth week for the female patient and at the beginning of the tenth week for the male patient. Neither of them suffered any general disease (except high blood pressure for the male patient and osteoporosis for the female patient). Both were treated with dentures made from Thermosens (under regular laboratory mode).

**Figure 10:**
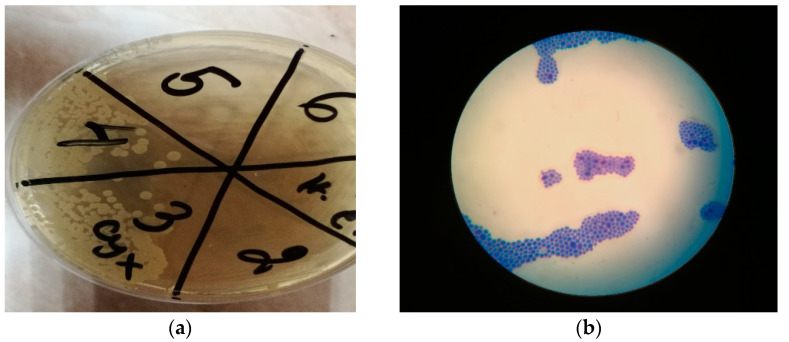
(**a**) SDA agar. Fungal growth on 3 (sample taken from the palate) and 4 (sample taken from the denture). No fungal growth observed on the negative control (n. c.). 2—sample from the vibrating line area, 5—sample from tuber maxillae sin., 6—sample from tuber maxillae dex. (**b**) Fungi, methylene blue stain, ×100 immersion oil microscopy.

After culturing of the samples on Sabouraud-dextrose agar (SDA), the present colonies were subsequently identified by MALDI-TOF MS as *Candida albicans*. (Figure 10a,b).

## 4. Discussion

The oral cavity is a habitat for microorganisms in large quantities and numerous varieties—pathogens, conditional pathogens, and saprophytes. The coarse and rough surface of dental prosthesis, the retention of food, and the constant humidity and temperature present suitable conditions for microbial contamination, colonization and propagation.

The surface characteristics of thermoplastic polymers exhibit numerous defects and a high level of roughness [12] that allow for the microbial colonization of their surface. Thermoplastic materials are challenging in terms of mechanical processing, making it difficult to produce a smooth and glossy surface [13]. The lack of this smoothness represents the optimal conditions [14] for the adhesion of microbial cells [15]. Although polyamide materials are characterized by a high level of mechanical properties, a modification [16] of the technological parameters [17,18] of their injection could be attempted to achieve an optimal texture. This modified surface needs to be resistant to impacts that would increase roughness [19,20] or deteriorate the quality of the material [20,21].

Surface modification could be a possible approach to identify surfaces that possess anti-biofilm properties [22]. The injection mode is precise and too short in duration, yet it depends on conditions and factors that could be manipulated, and the injection molding devices allow for it.

Attempting to improve the polyamide materials’ surface characteristics so that a surface with better anti-microbial [23] and bacterial attack inhibition effects is obtained, as well as a reduction in microbial activity [24,25,26,27,28,29,30], the authors altered some of the factors in the injection molding process. These factors and conditions are interdependent and mutually affecting.

With minimal alteration of the melting temperature (5 °С) and the pressure (0.5 Bar), no changes in terms of surface improvement were found in Biosens. What led us to apply variation of the temperature was the expectation that this would result in more even and more thorough melting of the material inside the machine tumbler. On the other hand, with a rise in temperature, the melt flow speed in the sprues changes as well, leading to a quicker filling of the mold, preventing uneven cooling of the material.

With minimal alteration to both the temperature (5 °C) and the pressure (0.5 Bar) in ThermoSens, the surface roughness of the material is significantly changed [31] in terms of roughness reduction [32]. This positive change in the surface texture is likely to result in: an improvement [33] in the mechanical strength and physical properties, a lack of microflora [34] or minimal changes [21], as well as a reduction in the conditions for colony formation [35,36]. To ascertain the presence or absence of such changes, further studies are necessary, including not only in vitro, but in vivo tests as well. A few volunteers are planned to be examined, treated with dentures manufactured using the modified laboratory protocol in a future study.

Ayaz et al. stated that striving for improvements in the texture of injection-molded materials is based on the fact that surface imperfection affects the adhesion and colonization of pathogenic microorganisms. Biofilm accumulation is the main factor in the etiology of denture stomatitis, emerging due to surface irregularities [37].

Verran and Maryan [38], Quirynen et al. [39], and Radford et al. [40] reported that dental materials on polyamide bases are rougher than PMMA materials. This statement is in agreement with Yunus et al. [41], Ucar et al. [42], and Kurkcuoglu et al. [43]. In their studies, they found a direct correlation between the surface roughness and adhesion of microorganisms. These findings correspond with some previous investigations of the authors of this article.

Kohli and Bhatia stated that the hydrophilic behavior of polyamide materials is due to the amide groups in their polymeric chain. Nylon, being hygroscopic, swells when immersed in a humid medium, increasing its irregularities [44].

Some substances, including saliva, alcohol, and acids produced by bacteria, may affect the structure and surface features of the restorations [45]. Arslan et al. assumed that material aging increases roughness and hydrophilicity [46]. Atalaya et al., in their study, declared that a smoother surface guarantees higher hydrophobicity and lower surface tension [47]. Liebermann et al. concluded that increased temperature and pressure of injection may change the polarity of the molecules, and that this can consequently cause alterations in the surface structure and wetting [48].

It is assumed that raised temperature leads to better and more even melting of the material, while increasing pressure leads to quicker and more uniform filling-up of the mold and therefore to its more uniform cooling down, both on the surface and internally. Both factors can reduce the cooling-induced tension on the surface and within the mold, and finally, this can cause the smoothing of the surface texture of the injected material.

## 5. Conclusions

By modifying the technological mode during injection molding, a smoother surface was achieved in one of the studied materials, and this variation could affect other factors and conditions during the process. Further studies should be conducted to find out whether such changes in the laboratory protocol affect the mechanical properties of these materials, and if so, in what range.

## Figures and Tables

**Table 1:** Materials, technological parameters, and manufacturer.

Material	Type	Time	Temperature	Pressure	System	Manufacturer
Perflex Biosens (BS)	Polyamide (MSDS: no declaration)	18 min	300 °C	8–9 Bar	Thermopress 400	Perflex, Israel
VertexTM ThermoSens (TS)	Polyamide (MSDS: no declaration)	18 min	290 °C	6 Bar	Vertex Thermoject 22	Vertex Dental B.V., The Netherlands

**Table 2:** Materials, modified technological parameters, and manufacturer.

Material	Type	Time	Temperature	Pressure	System	Manufacturer
Perflex Biosens (BS)	Polyamide (MSDS: no declaration)	18 min	305 °C	9.5 Bar	Thermopress 400	Perflex, Israel
VertexTM ThermoSens (TS)	Polyamide (MSDS: no declaration)	18 min	295 °C	6.5 Bar	Vertex Thermoject 22	Vertex Dental B.V., The Netherlands

**Table 3:** Materials, mode, and dimensions of the defects.

Material		Sample No.1 Length/Width (Microns)	Sample No.2 Length/Width (Microns)	Sample No.3 Length/Width (Microns)	Sample No.4 Length/Width (Microns)	Sample No.5 Length/Width (Microns)	Mean Value Length/Width (Microns)
	Mode						
Thermosens	Regular mode	15/18	12/12	20/14	15/12	16/15	15.6/14.2
	Modified mode	1/1	1.5/1	3/1	1.2/1	1/1	1.54/1
Biosens	Regular mode	20/25	23/21	15/14	21/20	28/25	21.4/21
	Modified mode	12/10	14/10	15/15	10/10	12/10	12.6/11
